# Genetic Modification of the Association between Peripubertal Dioxin Exposure and Pubertal Onset in a Cohort of Russian Boys

**DOI:** 10.1289/ehp.1205278

**Published:** 2012-10-10

**Authors:** Olivier Humblet, Susan A. Korrick, Paige L. Williams, Oleg Sergeyev, Claude Emond, Linda S. Birnbaum, Jane S. Burns, Larisa M. Altshul, Donald G. Patterson, Wayman E. Turner, Mary M. Lee, Boris Revich, Russ Hauser

**Affiliations:** 1 Department of Environmental Health, Harvard School of Public Health, Boston, Massachusetts, USA; 2 Center for Health and Community, University of California–San Francisco, San Francisco, California, USA; 3 Channing Laboratory, Department of Medicine, Brigham and Women’s Hospital and Harvard Medical School, Boston, Massachusetts, USA; 4 Department of Biostatistics, Harvard School of Public Health, Boston, Massachusetts, USA; 5 Department of Physical Education and Health, Samara State Medical University, Samara, Russia; 6 Chapaevsk Medical Association, Chapaevsk, Russia; 7 BioSimulation Consulting Inc., Newark, Delaware, USA; 8 National Institute of Environmental Health Sciences/National Cancer Institute, National Institutes of Health, Department of Health and Human Services, Research Triangle Park, North Carolina, USA; 9 Environmental Health and Engineering, Inc., Needham, Massachusetts, USA; 10 EnviroSolutions Consulting, Inc., Jasper, Georgia, USA; 11 Centers for Disease Control and Prevention, Atlanta, Georgia, USA; 12 Pediatric Endocrinology Division, Departments of Pediatrics and Cell Biology, University of Massachusetts Medical School, Worcester, Massachusetts, USA; 13 Center for Demography and Human Ecology, Institute for Forecasting, Russian Academy of Sciences, Moscow, Russia

**Keywords:** children, development, gene–environment interaction, PCBs, puberty, TCDD

## Abstract

Background: Exposure to dioxins has been associated with delayed pubertal onset in both epidemiologic and animal studies. Whether genetic polymorphisms may modify this association is currently unknown. Identifying such genes could provide insight into mechanistic pathways. This is one of the first studies to assess genetic susceptibility to dioxins.

Objectives: We evaluated whether common polymorphisms in genes affecting either molecular responses to dioxin exposure or pubertal onset influence the association between peripubertal serum dioxin concentration and male pubertal onset.

Methods: In this prospective cohort of Russian adolescent boys (*n* = 392), we assessed gene–environment interactions for 337 tagging single-nucleotide polymorphisms (SNPs) from 46 candidate genes and two intergenic regions. Dioxins were measured in the boys’ serum at age 8–9 years. Pubertal onset was based on testicular volume and on genitalia staging. Statistical approaches for controlling for multiple testing were used, both with and without prescreening for marginal genetic associations.

Results: After accounting for multiple testing, two tag SNPs in the glucocorticoid receptor (*GR/NR3C1*) gene and one in the estrogen receptor-α (*ESR1*) gene were significant (*q* < 0.2) modifiers of the association between peripubertal serum dioxin concentration and male pubertal onset defined by genitalia staging, although not by testicular volume. The results were sensitive to whether multiple comparison adjustment was applied to all gene–environment tests or only to those with marginal genetic associations.

Conclusions: Common genetic polymorphisms in the glucocorticoid receptor and estrogen receptor-α genes may modify the association between peripubertal serum dioxin concentration and pubertal onset. Further studies are warranted to confirm these findings.

Most diseases arise from the interplay between environmental factors and genetic susceptibility (Bookman et al. 2011). A substantial number of gene–environment interactions have been identified and replicated in epidemiologic studies (Hunter 2005). However, many environmental factors have yet to be assessed in gene–environment studies, including exposure to dioxins. Dioxins are a class of persistent environmental pollutants that have been linked to cancer (Michalek and Pavuk 2008), diabetes (Consonni et al. 2008), cardiovascular disease (Humblet et al. 2008), and endocrine disruption (Pavuk et al. 2003).

Different rat strains have lethal doses (median lethal dose, at 50%; LD_50_) of 2,3,7,8-tetrachlorodibenzo-*p*-dioxin (TCDD) that vary by a factor of 1,000 (Pohjanvirta et al. 1993). Dioxins may therefore be an environmental exposure for which genetic factors affect susceptibility. Identifying genetically susceptible subgroups is important for ascertaining the exposure levels that are sufficiently low to protect the most vulnerable members of the population, and may also clarify dioxin’s mechanisms of action.

Dioxin’s mechanism of action has been studied for > 30 years (Okey 2007), which facilitates the study of gene–environment susceptibility. Dioxins bind to the aryl hydrocarbon receptor (AhR), which, after dissociating from its complex of chaperone proteins in the cytoplasm (including HSP90 and XAP2) translocates to the nucleus. There it forms a complex with the ARNT protein, which binds to dioxin-responsive elements and alters the expression patterns of a large number of genes (Franc et al. 2008). In addition to its direct effect on transcription, the AhR is involved in many protein–protein interactions, including with various protein kinases, and pathways related to cell-cycle progression and apoptosis (Puga et al. 2009). Although the specific molecular mechanisms that transduce the toxic effects of dioxins are not fully understood, the pathways described above provide a ready list of potential genes that might affect susceptibility to dioxin exposure, thus allowing a targeted genetic approach.

There is currently no clear consensus over the optimal statistical method for analyzing medium- to large-scale gene–environment interaction studies (Thomas 2010) in order to minimize false positives due to testing many of these interactions. One common approach is to test all gene–environment interactions, followed by a suitably stringent adjustment for multiple comparisons (Le Marchand and Wilkens 2008; Van Hee et al. 2010). Another approach is to only test the gene–environment interactions among the subset of single nucleotide polymorphisms (SNPs) that have significant marginal genetic associations (Le Marchand and Wilkens 2008); however, this method may fail to detect gene–environment interactions where the genetic main effect is weak. Few studies have applied these two statistical approaches to a single epidemiologic data set in order to compare their results.

We used data collected as part of the Russian Children’s Study to evaluate potential gene–environment interactions that may affect the association of dioxin exposures with pubertal onset. The Russian Children’s Study has the primary aim of assessing pubertal maturation and growth in a prospective cohort of boys living in Chapaevsk, Russia, a town contaminated with dioxins and other chlorinated chemicals by past industrial activity. Boys were recruited at 8 or 9 years of age. The timing of pubertal onset was the outcome of interest in all analyses and was assessed at recruitment and during prospective yearly clinical examination, using both pubertal staging and testicular volume measurements. In recently published research from this cohort, peripubertal dioxin exposure has been linked to delayed pubertal onset in adolescent boys (Korrick et al. 2011), an association supported by numerous animal studies (Bell et al. 2007; Gray et al. 1995; Hamm et al. 2003). In this study we assessed whether genetic polymorphisms modified the association of the boys’ serum dioxin concentrations with pubertal onset, by selecting common polymorphisms in the well-characterized molecular pathway of biological response to dioxin exposure. This is one of the first large-scale attempts to assess genetic susceptibility to the health effects of dioxins.

## Methods

*Study population*. The Russian Children’s Study is an ongoing prospective cohort of 499 peripubertal boys (including seven sibling pairs) and their mothers in Chapaevsk, Russia. A total of 572 eligible boys 8 or 9 years of age were identified using the town-wide health insurance information system and recruited between 2003 and 2005 (Williams et al. 2010). The study was approved by the Human Studies Institutional Review Boards of the Chapaevsk Medical Association, Harvard School of Public Health, University of Massachusetts Medical School, U.S. Centers for Disease Control and Prevention (CDC), and Brigham and Women’s Hospital. Before participation, the parent or guardian signed an informed consent and the boy signed an assent form. Additional consent was received to conduct the genetic analyses.

At study entry, a physical examination was conducted and each boy and mother provided blood samples for analyses of lead, dioxins, and polychlorinated biphenyls (PCBs). A health, lifestyle, and diet questionnaire developed with Russian collaborators (Hauser et al. 2005; Lee et al. 2003) was administered by a nurse to each boy’s mother or guardian. A validated Russian Institute of Nutrition semiquantitative food frequency questionnaire was used to ascertain the child’s typical dietary intake over the previous year (Martinchik et al. 1998; Rockett et al. 1997) and to estimate total daily energy intake and distribution of energy from fat, protein, and carbohydrate.

*Physical examination*. At study entry and at annual follow-up visits, a standardized anthropometric examination and pubertal assessment was performed by a single study investigator (O.S.), according to a written protocol and without knowledge of the boys’ or mothers’ dioxin levels. Pubertal status was staged from 1 to 5 on the basis of visual inspection and comparison with published photographs, according to internationally accepted criteria (Marshall and Tanner 1970). Genitalia staging was assessed on the basis of the size and maturity of the genitalia. Testicular volume (TV) was measured using Prader beads (orchidometer). Two different measures of pubertal onset were considered: TV of > 3 mL for either testis, and genitalia stage 2 or higher (G2).

*Analysis of blood samples for dioxins, PCBs, and lead*. Blood samples were centrifuged and the serum was aliquoted and stored at –35°C until shipment on dry ice to the CDC for chemical analyses by the National Center for Environmental Health (Atlanta, GA, USA). Blood lead levels were measured using atomic absorption spectrometry (Williams et al. 2010). Chemical analyses for dioxins and PCBs were performed using high-resolution mass spectrometry (Humblet et al, 2010). Total cholesterol and triglycerides were measured enzymatically, and the serum total lipid content was calculated as by Phillips et al. (1989). All dioxin, furan, and PCB measurements were presented as lipid adjusted.

*Genotyping and SNP selection*. DNA for genotyping was obtained from whole blood. Genotyping was performed using the Illumina GoldenGate assay at the Applied Genomics Technology Center at Wayne State University (Detroit, MI, USA).

Forty-six genes and two intergenic regions were selected because of their potential relevance to either the AhR molecular pathway or to pubertal onset. Genes were selected that met at least one of the following criteria:

Genes known to play a key role in the AhR response to dioxin.
Genes identified by querying two public databases for proteins that interact with the AhR: Entrez Gene (National Library of Medicine; http://www.ncbi.nlm.nih.gov/gene), and STRING [European Molecular Biology Laboratory (Szklarczyk et al. 2011)].
Genes known to be induced by dioxins via AhR mechanisms (e.g., *CYP1A1*). Genes related to reproductive outcomes, including those recently identified from four genome-wide associations studies of pubertal onset (He et al. 2009; Ong et al. 2009; Perry et al. 2009; Sulem et al. 2009) or from mechanistic studies [i.e., *GPR54* and *KISS1* (Dedes 2012)], or suggested by a previous study of dioxin-related genes and reproductive abnormalities (Sone and Yonemoto 2008).

SNPs from these genes were identified using the HapMap Genome Browser (Phase 1 and 2, release 24; http://hapmap.ncbi.nlm.nih.gov/), among the CEPH (Caucasian) population. Tagging SNPs were used to parsimoniously represent the variability in clusters of correlated SNPs. Tag SNPs were selected so as to ensure a pairwise *R*^2^ > 0.8 with all SNPs with minor allele frequency > 0.1 within 10 kb proximal and distal of each candidate gene, using Haploview version 4.2 (Barrett et al. 2005). The final criterion was SNP compatibility with the Illumina GoldenGate technology as determined by the Illumina Assay Design Tool.

Four hundred eighty SNPs were genotyped. For approximately 5% of SNPs, the genotypes were manually reclassified because of quality control results indicating poor clustering. Then 67 (14%) were excluded because of either a low minor allele frequency (< 5%), or a call rate < 95%. An additional 76 (16%) were excluded because of a significant Bonferroni-adjusted chi-square test rejecting the assumption of Hardy–Weinberg equilibrium. After these exclusions, 337 SNPs were included in our statistical analyses.

The list of 46 genes and two intergenic regions from which SNPs were selected, along with the number of genotyped and included SNPs, is shown in Table 1. The list of all 337 included SNPs, along with descriptive information and minor allele frequencies, is shown in Supplemental Material, Table S1 (http://dx.doi.org/10.1289/ehp.1205278).

**Table 1:** Numbers of SNPs genotyped and passing the inclusion criteria, among 46 candidate genes and two intergenic regions.

Gene	Total tag SNPs	Tag SNPs passing inclusion criteria	Gene inclusion criteria
AHRR	27	24
AIP/ARA9/XAP2	2	0
ARNT	5	4	Canonical AhR
AhR	11	8
HSP90	6	4
p23/PTGES3	4	4
ARNTL	19	14
CCNT1	3	3
DAP3	3	1
ESR1/ER-a	49	33
GTF2F1	3	1
GTF2F2	9	6
HIF1A/MOP1	6	2
NCOA1/SRC1/RIP160	11	6
NCOA2/SRC2	19	14
NEDD8	4	4
NR2F1	2	2	AhR interacting genes (Entrez Gene)
NRIP1/RIP140	10	8
RB1	4	4
RELA/NFKB3	4	2
SMARCA4	10	6
SP1	4	4
SRC	14	10
TAF4	6	4
TAF6	6	4
TBP	6	3
XPO1	5	5
AR	2	0
CREB1	4	3
ERB/ESR2	16	12
GR/NR3C1	18	15
GSTCD	5	5	AhR interacting genes (STRING)
GSTM1	1	0
MYC	6	5
PPARA	16	11
RELB	6	4
UGT1A5	16	10
CYP1A1	2	1
CYP1A2	2	1	Dioxin-induced genes
CYP1B1	6	5
TP53	5	1
ARNT2	58	36	Genes associated with reproductive outcomes: Sone and Yonemoto 2008
CYP17A1	6	4	
9q31.2, region 1	12	10	Genes associated with reproductive outcomes (GWAS studies): He et al. 2009; Ong et al. 2009; Perry et al. 2009; Sulem et al. 2009
9q31.2, region 2	14	11	
LIN28b	13	10	
GPR54	3	2	Genes associated with reproductive outcomes (puberty-related)
KISS1	8	4	
Total	480	337
GWAS, genome-wide association studies. See “Methods” for additional information on gene selection.						

*Statistical analysis*. We considered longitudinal data on pubertal status from the initial entry visits and up to four annual follow-up visits. We evaluated associations using Cox proportional-hazards models for time to pubertal onset. Onset was defined as TV > 3 mL (either testis), or G2. The timing of onset was defined as the midpoint between the first visit at which onset was observed and the previous visit. Pubertal onset before enrollment was assumed to occur 6 months before enrollment, and boys who were still prepubertal at their last study visit were censored at that visit.

Dioxin toxic equivalents (TEQs; picograms per gram lipid) were computed on a lipid standardized basis using the 2005 toxic equivalency factors to weight each congener’s potency relative to TCDD (Van den Berg et al. 2006). We also calculated the summed concentrations of non-coplanar PCBs, including mono-*ortho*-substituted PCBs (ΣPCBs; nanograms per gram lipid). Both measures were log_10_-transformed for statistical analysis to improve normality.

In a previous study from this cohort (Korrick et al. 2011) a covariate selection process was carried out to identify significant predictors of pubertal onset as well as potential confounders of the association between peripubertal dioxin exposure and pubertal onset. Because genotypes are available only for a subset of the boys, we re-ran the previous multivariate model within this subset of boys for both measures of pubertal onset, and excluded two variables that did not have *p* < 0.10 in either model: household income and maternal alcohol consumption during pregnancy. We furthermore excluded four potential intermediates between genotype and pubertal onset, to avoid potential overadjustment (VanderWeele et al. 2012): son’s birth weight and gestational age, and peripubertal height and body mass index (BMI). The mothers’ age at menarche, a strong predictor of the boys’ age of pubertal onset in previous analyses (Humblet et al. 2011) was not included here, to avoid overadjustment by controlling for a predictor of the son’s genetic makeup. Finally, the son’s peripubertal ΣPCBs were included in all models because evidence suggests that they confound the association of peripubertal TEQs with pubertal onset (Korrick et al. 2011). The covariates included in the final model were parental education (maximum of maternal and paternal), son’s blood lead > 5 μg/dL (which was associated with later pubertal onset in this cohort) (Williams et al. 2010), diet at 8 or 9 years old (total calories and percent calories from protein, fat, and carbohydrate, respectively), and log_10_-transformed ΣPCBs.

To identify significant genetic interactions with the boys’ peripubertal TEQs, we fitted separate multivariate Cox models for each SNP, which included an interaction term consisting of the number of minor alleles (0, 1, or 2) multiplied by the boys’ peripubertal log_10_ TEQ value. We then controlled the false discovery rate (FDR) for the gene–environment interaction terms at *q* < 0.2 (Benjamini and Hochberg 1995) to adjust for multiple comparisons within each pubertal outcome of interest. The Bonferroni correction was also applied for comparison with the FDR results. As a sensitivity analysis, these models were re-run after including the four potential intermediates and the mother’s age at menarche.

For comparison, we also applied a second method for multiple comparison correction. We first prescreened all SNPs for significant (i.e., *p* < 0.05) main effects with pubertal onset (either TV or G2) in multivariate Cox models. Then, to limit the number of statistical tests, we tested interaction terms between dioxin TEQ values and SNPs with a significant main effect only.

Finally, to explore the shape of the dose-response curves, the models were re-run after including five indicator variables for tertiles of dioxin within two genetic groups (i.e., those with 0 minor alleles, and those with 1 or 2 minor alleles). The reference group consisted of those in the lowest dioxin tertile and with 0 minor alleles.

All statistical analyses were conducted using SAS version 9.2 (SAS Institute Inc., Cary, NC, USA), except for the Hardy–Weinberg equilibrium test, which used Plink version 1.07 (Purcell et al. 2007).

## Results

*Population descriptive information.* Of the 499 boys in the full cohort, 10 were excluded from puberty-related analyses due to chronic medical conditions. Of the remaining 489, 408 (83%) were included in the genotyping analysis as a result of consenting to participate in the genetic portion of the study and also having sufficient blood available to genotype. Demographic characteristics are shown in Table 2. Of these, 392 (96%) were included in the final analyses because they had complete covariate data. For each of the 392 boys, > 90% of SNPs were successfully genotyped. For 95% of SNPs there were three or fewer individuals with missing genotypes. For quality control, one sample of reference DNA was analyzed on each of the five plates of samples: 98.4% of nonmissing genotypes were identical in all five samples.

**Table 2:** Demographic characteristics among 408 boys.

	Mean ± SD	n (%)
Characteristics at study entry		
Age (years)	8.4 ± 0.5
Height at study entry (cm)	130 ± 6.2
Weight at study entry (kg)	27 ± 5.5
BMI at study entry (kg/m2)	16 ± 2.3
Blood lead (≥ 5 µg/dL)		111 (27)
Proportion of dietary fat (%)	34 ± 6.0
Proportion of dietary protein (%)	12 ± 1.5
Household characteristics		
Low parental education (secondary education or less)		25 (6.4)
Missing information: proportion of dietary fat (n = 1), proportion of dietary protein (n = 1).			

This analysis included follow-up of up to four annual visits after the baseline health examination when the boys were 8 or 9 years old. The proportion of boys with pubertal onset by the end of follow-up was 90% for TV and 93% for G2. Detailed information on pubertal progression in this cohort has been published previously (Humblet et al. 2011; Korrick et al. 2011).

The median peripubertal value for total dioxin TEQs was 21 pg TEQ/g lipid, and for ΣPCB concentration was 197 ng/g lipid. Detailed information on the serum concentrations of dioxins, furans, and PCBs among participants in this cohort has been published previously (Burns et al. 2009; Humblet et al. 2010).

*Associations of dioxins with pubertal onset, without gene–environment interactions.* The association of the boys’ peripubertal total dioxin TEQs with the two measures of pubertal onset age (based on TV > 3 mL and G2) was assessed in analyses adjusted for other covariates (serum PCBs, lead and diet at age 8–9 years, and parental education). A 10-fold increase in serum total TEQ was associated with a hazard ratio of 0.46 (95% CI: 0.23, 0.95; *p* = 0.04) for TV pubertal onset, and 0.46 (95% CI: 0.26, 0.98; *p* = 0.04) for G2. Hazard ratios < 1 indicate that dioxin exposure was associated with later pubertal onset.

**Figure 1:**
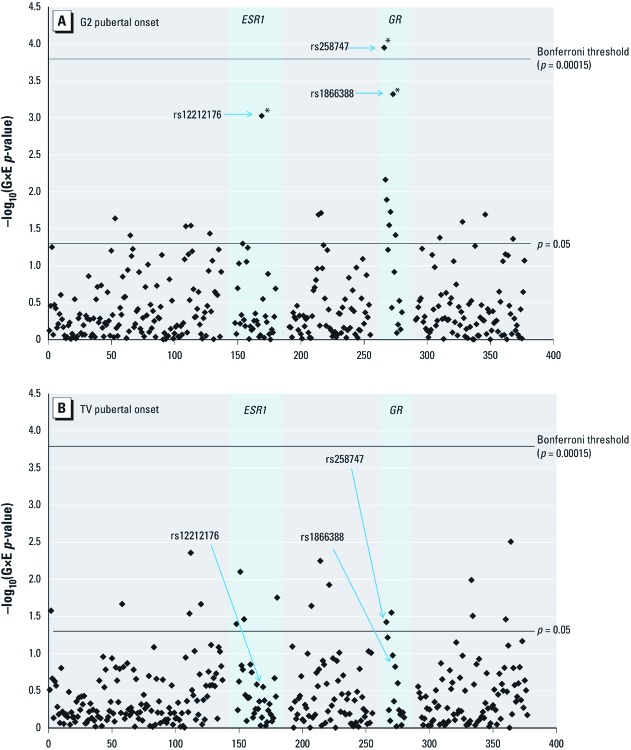
Manhattan plots: statistical strength of association (–log_10_ *p*-value) for the gene–dioxin interaction terms of all 337 SNPs (*x*-axes), for both G2 (*A*) and TV (*B*) pubertal onset. Statistical strength of interaction (–log_10_ *p*-value) is plotted against genomic position within the 46 genes and 2 intergenic regions. The number of SNPs with gene–environment (G×E) *p*-value < 0.05 was 19 for G2 and 18 for TV, with 17 expected by chance for each outcome. The two highlighted genes (*ESR1* and *GR*) are those containing SNPs with G×E FDR *q* < 0.2 for either G2 or TV. The Bonferroni threshold is calculated as 0.05/337 = 0.00015. *SNPs with G×E FDR *q* < 0.2.

*Gene–environment interactions.* Manhattan plots for the –log_10_-transformed gene–environment interaction *p*-values of all 337 SNPs are shown in Figure 1, for both G2 (Figure 1A) and TV (Figure 1B). Three SNPs were found to have interactions that were robust to adjustment for multiple comparisons, as indicated by a *q*-value < 0.2: two in the glucocorticoid receptor gene (*GR/NR3C1,* rs258747 and rs1866388) and one in the estrogen receptor-α gene (*ESR1*, rs12212176), all of them for G2 pubertal onset (Figure 1). The gene–environment interaction for rs258747 (*GR/NR3C1*) was also statistically significant using the more conservative Bonferroni adjustment (Figure 1A). The two *GR/NR3C1* SNPs listed above are part of a cluster of SNPs within this gene that are in high linkage disequilibrium, and whose gene–environment interaction terms have similarly strong associations with the outcome. In contrast, rs12212176 (*ESR1*) was the only SNP within the estrogen receptor-α gene with a gene–environment *p*-value < 0.05.

For TV pubertal onset, none of these three SNPs’ gene–environment *q*-values were < 0.2, although for rs258747 the *p*-value was < 0.05 (Figure 1; Table 3). Table 3 shows the pubertal onset hazard ratios for a 10-fold increase in the boys’ serum dioxin TEQ within each genotype for these three SNPs, for both G2 and TV pubertal onset, from models with an additive gene–environment interaction term. Although the gene–environment interactions are weaker for TV than for G2, for both outcomes there are similar patterns of significant TEQ–pubertal onset hazard ratios among the homozygous major subgroups of these three SNPs (with hazards ratios < 1; i.e., higher serum TEQs associated with delayed pubertal onset). In contrast, for G2 pubertal onset, in the homozygous minor subgroup of all three SNPs the TEQ–pubertal onset hazard ratios were in the opposite direction (i.e., > 1), although this was statistically significant only for rs258747. For TV pubertal onset, in the homozygous minor subgroup of all three SNPs the TEQ–pubertal onset hazard ratios were slightly less than 1, and not statistically significant (Table 3). The gene–environment results for all SNPs are shown in Supplemental Material, Table S2 (http://dx.doi.org/10.1289/ehp.1205278). In sensitivity analyses adjusted for the four potential intermediates as well as maternal age at menarche, the results were similar to the models in which they were not adjusted for (data not shown).

**Table 3:** Association of log10 TEQs with both G2 and TV pubertal onset, by genotype, for SNPs with gene–environment interaction FDR q < 0.2 for either G2 or TV.

Gene	Tag SNP	Pubertal onset measure	Genotype	n (%)	TEQ pubertal onset HRa (95% CI)	
G2 pubertal onset											
GR	rs258747	G2	AA	119 (30)	0.15 (0.06, 0.38)
	p G×E = 0.0001*		AG	177 (45)	0.45 (0.23, 0.89)
			GG	96 (24)	1.36 (0.59, 3.14)
GR	rs1866388	G2	AA	226 (58)	0.23 (0.10, 0.51)
	p G×E = 0.0005*		AG	147 (38)	0.74 (0.36, 1.49)
			GG	19 (4.9)	2.39 (0.79, 7.20)
ESR1	rs12212176	G2	GG	270 (69)	0.32 (0.15, 0.68)
	p G×E = 0.0009*		GA	115 (29)	1.19 (0.52, 2.74)
			AA	7 (1.8)	4.38 (1.05, 18.26)
TV pubertal onset											
GR	rs258747	TV	AA	119 (30)	0.25 (0.10, 0.63)
	p G×E = 0.04		AG	177 (45)	0.46 (0.22, 0.94)
			GG	96 (24)	0.84 (0.34, 2.08)
GR	rs1866388	TV	AA	226 (58)	0.34 (0.15, 0.78)
	p G×E = 0.15		AG	147 (38)	0.56 (0.26, 1.22)
			GG	19 (4.9)	0.94 (0.28, 3.14)
ESR1	rs12212176	TV	GG	270 (69)	0.39 (0.18, 0.85)
	G×E p = 0.28		GA	115 (29)	0.61 (0.25, 1.49)
			AA	7 (1.8)	0.95 (0.21, 4.25)
Abbreviations: G×E, gene–environment interaction; HR, hazard ratio. aHazard ratio for pubertal onset per 10-fold increase in dioxin TEQs, in model with additive genotypes. All models adjusted for lead and PCB serum concentration and diet (total calories, % calories from protein, fat, and carbohydrate) at 8–9 years of age, and parental education. *FDR q < 0.2.											

To explore the shape of the dose–response curves, we modeled the interactions with five indicator variables, representing the three tertiles of serum TEQs, stratified into two genetic subgroups (i.e., 0 minor alleles vs. 1 or 2 minor alleles). The reference group consists of those in the lowest TEQ tertile and with 0 minor alleles. This analysis (Table 4) shows that the largest difference in the magnitude of the G2 hazard ratios between the two genetic subgroups of the *GR* SNPs was seen in the lowest tertile of TEQs.

**Table 4:** Association of TEQ tertiles with both G2 and TV pubertal onset, among those with 0 vs. 1 or 2 minor alleles, for SNPs with gene–environment interaction FDR *q* < 0.2 for either G2 or TV.

Outcome and SNP	TEQ tertiles	0 minor alleles	1 or 2 minor alleles
		HR (95% CI)	HR (95% CI)
G2 pubertal onset
rs258747 (GR)
G2, p G×E = 0.0001a*	1	1 (Reference)	0.57 (0.39, 0.84)
	2	0.39 (0.25, 0.62)	0.52 (0.35, 0.79)
	3	0.31 (0.17, 0.55)	0.41 (0.25, 0.66)
rs1866388 (GR)
G2, p G×E = 0.0005a*	1	1 (Reference)	0.47 (0.33, 0.68)
	2	0.46 (0.32, 0.66)	0.54 (0.38, 0.79)
	3	0.36 (0.23, 0.57)	0.45 (0.28, 0.72)
rs12212176 (ESR1)
G2, p G×E = 0.0009a*	1	1 (Reference)	0.71 (0.47, 1.06)
	2	0.62 (0.45, 0.86)	0.76 (0.52, 1.11)
	3	0.50 (0.34, 0.74)	0.84 (0.52, 1.37)
TV pubertal onset
rs258747 (GR)
TV, p G×E = 0.04a	1	1 (Reference)	0.72 (0.49, 1.06)
	2	0.49 (0.31, 0.78)	0.69 (0.46, 1.04)
	3	0.44 (0.25, 0.79)	0.43 (0.26, 0.70)
rs1866388 (GR)
TV, p G×E = 0.12a	1	1 (Reference)	0.74 (0.51, 1.07)
	2	0.58 (0.40, 0.83)	0.82 (0.56, 1.19)
	3	0.50 (0.32, 0.79)	0.43 (0.27, 0.71)
rs12212176 (ESR1)
TV, p G×E = 0.28a	1	1 (Reference)	0.88 (0.59, 1.31)
	2	0.68 (0.49, 0.95)	0.88 (0.60, 1.29)
	3	0.56 (0.37, 0.85)	0.49 (0.29, 0.82)
Abbreviations: G×E, gene–environment interaction; HR, hazard ratio. aGene–environment p-values shown here were calculated in models with additive genotype coding (as shown in Table 3), not in the model with tertile indicators presented here. In each model the reference group consists of the boys in the lowest TEQ tertile and with 0 minor alleles. All models adjusted for lead and PCB serum concentration and diet (total calories, percent calories from protein, fat, and carbohydrate) at 8–9 years of age, and parental education. *FDR q < 0.2.								

*Alternate statistical method: prescreening for marginal genetic associations before assessing gene–environment interactions*. We hypothesized that a similar set of SNPs would be identified by assessing gene–environment interactions only within the subset of SNPs with significant gene-only associations with the outcome. However, this was not the case. There were 23 SNPs whose additive genetic associations with TV pubertal onset were significant at *p* < 0.05 (in models without a gene–environment interaction term), and 17 such SNPs for G2; however, none had *q* < 0.2 for the main effect on pubertal onset [see Supplemental Material, Table S3 (http://dx.doi.org/10.1289/ehp.1205278)]. The gene–environment interactions were assessed for those SNPs with a main-effect *p* < 0.05, but none of these had G×E *q* < 0.2. The only gene–environment interactions with *p* < 0.05 among this subset were rs2881766 in *ESR1* for TV pubertal onset (*p* = 0.04), and rs11905013 in *SRC* for G2 pubertal onset (*p* = 0.02) (data not shown). None of the three SNPs with gene–environment *q* < 0.2 for G2 in our primary analysis (*GR* rs258747, *GR* rs1866388, and *ESR1* rs12212176) were detected using the present strategy, because they all had nonsignificant additive main-effect *p*-values for G2, even without adjustment for multiple comparisons: *p* = 0.98, *p* = 0.25, and *p* = 0.33, respectively [see Supplemental Material, Table S3 (http://dx.doi.org/10.1289/ehp.1205278)].

## Discussion

*Summary*. In this study of Russian adolescent boys we assessed whether the association of dioxin exposure with pubertal onset varied in boys with different genotypes. Serum total TEQ concentration was used to summarize dioxin exposure. Candidate genes were selected for relevance to either the AhR molecular pathway or to pubertal onset, and a representative group of polymorphisms was measured in each gene (i.e., tag SNPs). Our primary finding was that three SNPs (two from the glucocorticoid receptor gene, one from estrogen receptor-α) had gene–environment interactions robust to correction for multiple comparisons. The two significant tag SNPs in the glucocorticoid receptor were part of a cluster of nearby SNPs that all appeared to reflect a common association signal, which may indicate a more robust association (Soler Artigas et al. 2012). However, in additional analyses using an alternate statistical method of screening for gene–environment interactions, these three SNPs were not found to be significant. Furthermore, these interactions were significant only according to the primary analysis for pubertal onset based on genital staging, not for testicular volume.

Although the biologic mechanisms underlying these two measures of pubertal onset are not identical [i.e., genital maturation is dependent primarily on androgens, whereas testicular growth also requires gonadotropins in addition to androgens (Macleod et al. 2010; Raivio et al. 2007)], they are tightly correlated, and it was unexpected that some SNPs would have significant gene–environment interactions for one measure and not the other. Further study will be needed to determine whether this apparent difference is real, or if it instead is due to chance.

*Limitations*. First, the only outcome assessed in this study was pubertal onset among boys. The relevance to pubertal onset among girls, or to other dioxin-related outcomes, is unknown. Because the association between dioxins and male pubertal onset may be mediated through different mechanisms than for other outcomes (e.g., cancer), it remains to be determined whether our findings, if replicated, are applicable to other health outcomes (or to females). Second, this type of genetic study involves several hundred statistical comparisons, raising the possibility of false positives despite the statistical methods used to control for multiple comparisons. Third, this was a candidate gene study that did not assess the whole genome, so important genes may have been omitted. Fourth, even though tag SNPs were selected so as to ensure a pairwise *R*^2^ > 0.8 with all selected SNPs in the included genes, some tag SNPs did not pass the genotyping quality control criteria, leading to a reduction in the actual coverage.

Fifth, because we used tag SNPs instead of sequencing all genetic loci, it is likely that any causal genetic loci were unmeasured. The SNPs tagged by the three significant tag SNPs (7 SNPs for *GR* rs258747, 10 for *GR* rs1866388, and none for *ESR1* rs12212176) are listed in Supplemental Materials, Table S4 (http://dx.doi.org/10.1289/ehp.1205278). Sixth, our sample size provided limited power to analyze the gene–environment interactions of SNPs with moderate minor allele frequencies. Because of this limited power we also excluded SNPs with low minor allele frequencies. Seventh, the serum dioxin concentrations in this population were substantially higher than in other recent general population studies (Burns et al. 2009), which made it difficult to investigate associations with very low exposures. The median serum total 2005 TEQs of the 8- to 9-year-old Chapaevsk boys was three times the geometric mean from the U.S. National Health and Nutrition Examination Survey for males 12–19 years of age (there were no data on children < 12 years of age) (Patterson et al. 2008). Finally, an important assumption of this study is that the additively coded number of minor alleles for individual common SNPs is an appropriate marker of biologic susceptibility to environmental factors, which may not be the case.

*Potential biological mechanisms*. The glucocorticoid receptor (*GR/NR3C1*) potentiates the TCDD-induced expression of CYP1A1 in human aorta endothelial cells (Celander et al. 1997), although in another study this effect was seen only in rat H4IIe cells and mouse Hepa 1c1c7 cells, but not in human HepG2 or T47D cells (Sonneveld et al. 2007). Evidence for AhR–glucocorticoid receptor regulatory cross-talk has been reported in human HepG2 cells (Dvorák et al. 2008). *ESR1*, also known as the gene for estrogen receptor-α, can be either negatively or positively regulated by AhR ligands (Matsumura 2009), and furthermore is known to modulate dioxin-induced gene expression (Beischlag and Perdew 2005).

## Conclusions

This study was the first to assess genetic interactions with dioxin exposure, to our knowledge. It represents a potentially promising model for gene–environment interaction discovery. We chose an environmental exposure (serum dioxin concentration) for which substantial mechanistic information is available for selection of candidate genes. We collected information on multiple related outcomes, which allowed us to judge the plausibility of any genetic findings by assessing their consistency. The primary outcome was pubertal onset, an event experienced during the follow-up period by almost all our study participants, thus increasing the power of our analyses and also allowing the use of a prospective study design, removing any issues of retrospective exposure assessment.

We found three SNPs that were robust to the adjustment for multiple comparisons. However, there was less similarity than expected between the gene–environment interactions for the two measures of pubertal onset, and the results were sensitive to the choice of statistical method. Given the poor track record of replicating results from candidate gene studies, repeating these analyses in other studies will be necessary.

## Supplemental Material

(49 KB) PDF

(123 KB) XLSX
