# Supplementary material for: Genetic Modification of the Association between Peripubertal Dioxin Exposure and Pubertal Onset in a Cohort of Russian Boys
Source: Environ Health Perspect. 2012 Oct 10;121(1):111–7. doi: 10.1289/ehp.1205278 (PMC3546349; doi:10.1289/ehp.1205278)
Supplement: (49 KB) PDF [file ehp.1205278.s001.pdf]

SUPPLEMENTAL MATERIAL

Genetic Modification of the Association between Peripubertal Dioxin Exposure and Pubertal Onset in a Cohort of Russian Boys

Olivier Humblet, Susan A. Korrick, Paige L. Williams, Oleg Sergeyev, Claude Emond, Linda S. Birnbaum, Jane S. Burns, Larisa M. Altshul, Donald G. Patterson Jr., Wayman E. Turner, Mary M. Lee, Boris Revich, Russ Hauser

Supplemental Material Table of Contents:

- Table S1: Descriptive data on 337 included tag SNPs ………See separate Excel file
- Table S2: Gene x Environment interaction results of all SNPs, for both G2 and TV pubertal onset…………………………………………………See separate Excel file
- Table S3: Genetic associations with pubertal onset of all SNPs, in additive models, for both G2 and TV pubertal onset……………………………See separate Excel file
- Table S4: List of SNPs tagged with R^2 >0.8 by the 3 SNPs with significant (FDR q<0.2) gene-environment interactions………………………………………………p.2

SUPPLEMENTAL MATERIALS, TABLE S4: List of SNPs tagged with R^2 >0.8 by the 3 SNPs with significant (FDR q<0.2) gene-environment interactions

1. rs258747 (*GR*): 7 other SNPs tagged
   a. rs4634384
   b. rs852980
   c. rs258763
   d. rs10041520
   e. rs33383
   f. rs6877893
   g. rs33388

2. rs1866388 (*GR*): 10 SNPs tagged
   a. rs2963149
   b. rs2918416
   c. rs860457
   d. rs852982
   e. rs10052957
   f. rs852977
   g. rs258813
   h. rs190488
   i. rs258750
   j. rs2918417

3. rs12212176 (*ESR1*): 0 other SNPs tagged
